# A review of the psychological and familial perspectives of childhood obesity

**DOI:** 10.1186/2050-2974-1-7

**Published:** 2013-02-25

**Authors:** Yael Latzer, Daniel Stein

**Affiliations:** 1 Faculty of Social Welfare & Health Sciences, Haifa University, Haifa, Israel; 2 Eating Disorders Clinic, Psychiatric Division, Rambam Medical Center, Haifa, Israel; 3 Pediatric Psychosomatic Department, the Edmond and Lily Safra Children’s Hospital, The Chaim Sheba Medical Center, Tel Hashomer, affiliated with The Sackler Faculty of Medicine, Tel Aviv University, Tel Aviv, Israel

**Keywords:** Adolescence, Childhood, Familial obesity, Overweight, Psychological psychosocial

## Abstract

Childhood obesity is on the rise in both industrialized and developing countries. The investigation of the psychosocial aspects of childhood obesity has been the focus of long- standing theoretical and empirical endeavor. Overweight in children and adolescents is associated with a host of psychological and social problems such as reduced school and social performance, less favorable quality of life, societal victimization and peer teasing, lower self-and body-esteem, and neuropsychological dysfunctioning. Whereas community samples of obese youngsters usually do not show elevated psychopathology, clinically-referred overweight children show elevated depression, anxiety, behavior problems, attention deficit hyperactivity disorder and disordered eating. Parents’ perceptions of their child’s overweight highly influence the well-being of obese children and the way in which they perceive themselves.

The present review paper aims to broaden the scope of knowledge of clinicians about several important psychosocial and familial dimensions of childhood obesity: the psychosocial functioning, self and body esteem and psychopathology of overweight youngsters, the influence of children’s perceptions of overweight, including those of the obese children themselves on their well being, and the influence of parental attitudes about weight and eating on the psychological condition of the obese child.

## Review

The investigation of the psychological and familial aspects of childhood obesity has been the focus of long-standing theoretical and empirical effort [1,2]. In this review paper we aim to describe the main psychosocial dimensions of childhood obesity: overall psychosocial functioning, cognitive performance, self and body esteem and psychopathology in overweight youngsters, the influence of children’s perception of overweight on their well being, and the influence of parental attitudes about weight and eating on the psychological condition of the obese child.

The current review focuses on an updated analysis of the most relevant psychiatric and psychosocial issues in childhood overweight. In order to identify the relevant articles on this topic, we conducted a comprehensive systematic computerized literature search of the Cochrane, PUBMED, PSYCHLIT, PSYCHINFO, and ERIC databases from 1991–2012 using the following terms: “childhood obesity,” “childhood overweight” “pediatric obesity” “pediatric overweight” “overweight and obesity in youngsters and in adolescents,”. We also combined the terms: “overweight” and “obesity’ with “family”, “familial”, “prevalence”, “psychoeducation”, “psychiatric”, “psychopathology”, “psychosocial”, and “sociocultural”. Lastly, in order to provide a more clinically oriented review, we included in our analysis not only controlled and randomized controlled trails (RCTs), but also findings of open studies.

### Definitions

Obesity is a well-defined term in adults but in children and adolescents its definition is less consistent. The US Center for Disease Control (CDC) growth charts include gender-specific body mass index (BMI)-for age growth charts for ages 2 to 19 years [3]. Obesity among individuals 2–19 years old is defined as the 95th percentile or greater of BMI–for age, and overweight is defined as the 85th percentile or greater, but less than the 95th percentile of BMI-for age [3]. Because of the likelihood of stigmatization associated with the term “obesity”, several leading authorities in the field have suggested to define a “risk for overweight” as BMI between 85-95%, and overweight as a BMI > 95% (see for example [4,5]. An international forum of consensus development, the International Obesity Task Force (IOTF) [6], has recently recommended adopting the risk-related cut-off concept of Cole et al. [7], who developed age and sex specific cut-off points using the BMI. These charts extrapolate risk from the adult experience to children. The IOTF defines childhood overweight as a BMI of approximately 91% or greater and obesity as a BMI of approximately 99% or greater [6].

### Prevalence

Childhood obesity is epidemic around the globe, and has increased in prevalence over the past 2 decades in both industrialized and developing countries. Thus, whereas the prevalence of child and adolescent obesity has been relatively stable during the 1960th and early 1970th, it has begun to accelerate since the late 1970th [8,9]. Accordingly, in the US, the prevalence of overweight has doubled among youngsters 6 to 11 years of age and tripled among those 12 to 17 years of age between 1976–1980 and 1999–2002, respectively [8]. The prevalence of overweight in the United States around the end of the 1990th has been estimated around 10.4%, 15.3% and 15.5% for children 5–10, 6–11, and 12–19 years old, respectively [10]. In 2007–2008, 9.5% of American infants and toddlers have been at or above the 95th percentile of the weight-for-recumbent-length growth charts. Among American children and adolescents aged 2 through 19 years, 11.9% have been at or above the 97th percentile of the BMI-for-age growth charts; 16.9% have been at or above the 95th percentile; and 31.7% at or above the 85th percentile of BMI for age [11].

In the UK, the rate of overweight, including obesity, has risen from 17 to 22% and from 20 to 25% in 2–5 years old boys and girls, respectively, between the early 1990th and early 2000th [12]. Similarly high rates of childhood obesity have been also found recently in other Western countries. Thus, a recent review summarizes data showing that in the beginning of the 21st century, for the European region as a whole, around 20% of children aged 5–9 years are overweight (of which 6% are obese), and 16% of children aged 13–17 years are overweight (of which 4% are obese) [13]. Moreover, data from 11 countries of the European Union show that the annual increases in prevalence of overweight (including obesity) have risen from typically below 0.5 percentage points in the 1980s, to over 1.0 percentage points in the late 1990s, and it is still in the rise in the first years of the 21st century [13].

High rates of pediatric obesity have been observed also in non-Western countries in recent years. For example, in China, the overall prevalence of overweight and obesity for children aged 2–6 years has been 10.7% and 4.2%, respectively in the first years of the 21st century [14]. Interestingly, studies in some countries have found stabilization in the rate of pediatric overweight and obesity in recent years. This has been noted, for example, in the USA from 2000 to 2008 [11], and in France from 2000 to 2007 [15]. Still it is predicted that by the year 2025, childhood obesity will be the world’s number one health problem [16].

Genetic factors account for around 50% of the risk for overweight and obesity among children [17], whereas specific genetic or endocrine diseases account for less than 10% of all cases, and hypothalamic injuries and drug-induced obesity are even less common. As the gene pool has likely not changed during the past 2–3 decades, environmental, social and psychological factors are largely thought to account for the high prevalence of overweight and obesity in recent years. It is important to note that while the individual effect sizes of most environmental factors are likely to be small and the validation of their causality is not straight-forward [17], the simultaneous change in many socio-environmental risk factors from the second half of the 20th century may explain the parallel changes in the prevalence of childhood obesity [10,18].

### The psychosocial perspective

#### Educational achievements

Overweight in children and adolescents may be associated with a host of psychological and social problems, which can have a considerable deleterious impact on the psychological development and quality of life of the overweight youngster. In addition, the school and social performance of obese children are often [19,20], although not always [21,22], less favorable in comparison to normal weight youngsters. One explanation for this finding is that overweight children are absent form school significantly more than normal-weight children. Thus, the obese category remains a significant contributor to the number of days absent from school even after adjusting for age, race/ethnicity, and gender [23]. Obese children are in addition at increased risk of psychosocial distress relative to their non-obese peers [24], and their overall quality of life [20,25,26] and emotional well-being [27,28] are often less favorable. Lastly, lower school performance during childhood and adolescence is an important predictor for the development of obesity in later life [29]. Psychosocial factors such as global self-concept, body dissatisfaction [25] and weight-based teasing [19,30], may mediate in the influence of overweight/obesity on the performance of children and adolescents in their required age-specific developmental tasks and their well-being.

Interestingly, lower childhood IQ scores may be associated with an increased prevalence of adult obesity, this relation largely mediated via lower educational achievements and less adoption of healthy diets in later life [31]. From a different perspective, children and adolescents whose diets are nutritious and whose participation in physical activity is high have been found to perform better on various measures of cognitive performance and academic achievement [32]. Thus, in one cross- sectional study of school children in Iceland, body mass index (BMI), diet and physical activity have explained up to 27% of the variance in academic achievement when controlling for gender, parental education, family structure, absenteeism, depressed mood, and self-esteem [32]. Moreover, an association has been recently found between overweight/obesity and decreased visuospatial organization in 8–16 years old [21], and reduced motor, verbal, cognitive and social skills in 4–9 years old [33], and even in 2–3 years old [34] children, particularly, although not exclusively, in boys. The finding of less than normal visuospatial functioning in obese youngsters retains its significance after adjusting for confounding variables such as parental/familial characteristics, sports participation, physical activity, hours spent watching TV, psychosocial development, blood pressure, and serum lipid profile [21]. Although these studies do not provide causal explanations for their findings. The liklihood of the reduced skill attainment during infency and childhood to decrease the academic performance of overwiet adolescents and adults is of considereble concern ([33,34]).

#### Self and body esteem

The association between overweight and global self-esteem in youngsters is not straightforward. Thus, some studies have indicated that overweight youngsters have lower self-esteem than non-obese teens [7,21,22,35-39], whereas other studies have shown no difference in the self-esteem of overweight and normal weight youngsters [1,40,41]. Still other studies have found that self-esteem is no longer lower in overweight children when controlling for the influence of body image and body dissatisfaction [42].

Self-esteem in overweight children and adolescents varies with gender, with females; may be at a greater risk of developing low self-esteem than males [43,44]. The influence of age on self-esteem is not clear cut. Thus, some studies have shown that young overweight children are at particular risk for lower self-esteem [27], whereas in others, adolescents are more vulnerable than younger children [42,43].

The association between the severity of overweight and self-esteem in overweight and obese children is not straightforward. Still, although some studies have shown no association in obese youngsters between BMI levels and self-esteem [43] and others have suggested that many obese youngsters assessed in the community are not concerned with their weight [9], most studies do show a greater concern with weight in obese vs. normal weight children and adolescents, likely influencing their sense of self-esteem [45-47].

The socio-cultural status and region of residence may also have an influence on self-esteem. For example, overweight may influence self-esteem in Caucasian and Latin American but not in African-American girls) [2,48], or in Australian as opposed to Hong Kong youngsters [41]. In addition, low self-esteem in overweight youngsters may be related to the presence of disturbed eating [49], dieting behavior, and preoccupation with weight and shape [22,42,48], and to an overall inactive lifestyle [22], particularly among overweight females, these being in some cases even more influential than the actual overweight. Lastly, bullying has been found a worldwide factor in decreasing the sense of self-esteem of obese youngsters, irrespective of their age and gender [49].

The findings concerning the association of body image with overweight are more consistent in comparison to self-esteem. Favorable body image and perception have been found to be inversely correlated with the actual weight, and overweight youngsters usually show more body image disturbances and more body-related negative attitudes than their normal weight counterparts [1,2,38,41,50-52]. In some studies [21,38,42], body dissatisfaction has been found to mediate the association between obesity and self-esteem, with obese children with greater body dissatisfaction having significantly lower self-esteem. Still other studies have shown no mediating effect of body image on self-esteem [41].

#### Children’s perceptions of childhood obesity

Adiposity is very visible to youngsters, and even young children tend to rate disease and minor deformities as less detrimental than obesity, and to ascribe more negative attitudes to overweight than normal-weight youngsters [5]. Studies assessing attributes related by children to obesity emphasize primarily those of laziness, selfishness, lower intelligence, social isolation, poor social functioning and low academic success [50]. Overweight children are also perceived by their normal weight peers as being less healthy, eating less well, and exercising significantly less [50]. This stigmatization may stem from children’s beliefs that weight is under one’s own personal control [5].

In addition, children as young as five years old are very aware of their own overweight, which has a considerable influence on the more derogatory manner in which they perceive their own appearance, athletic ability, physical competence, social competence, self-worth, and general health [5,28,53-55]. These findings support the notion that independent of their own weight status or gender, children share the overall negative parental and societal perceptions towards those who are overweight or obese.

Considering the issue of popularity, overweight preadolescent girls are significantly less likely to be considered pretty, although they may not differ in popularity [56]. A considerable decrease in popularity occurs during adolescence, as weight teasing in these age groups is highly correlated with actual weight. Weight-related teasing is greater in girls than in boys and in adolescents than in younger children [57]. Moreover, overweight girls may report more victimization compared with their average-weight peers and are less likely to date than their peers [5,58]. Similarly, overweight children and adolescents are often less liked, and less chosen as friends, and are more rejected, isolated and peripheral to social networks than their normal-weight peers [5,59]. Indeed, large scale community-based studies have shown a greater prevalence of overweight and obesity in preschool children showing problematic relations with their peers in comparison to children with adaptive peer relationships [60].

There is currently growing literature showing that overweight and obese children and adolescents are targets of societal stigmatization and teasing by peers, educators, and even parents [5,16,19,30,56,57,61-63]. Puhl and Latner [5] have recently critically analyzed the findings about the association between obesity, teasing, and health among youth. According to these authors and others [63], higher BMI is associated with more frequent and intense stigmatization in children and adolescents of both sexes. Internalization of this weight stigma in overweight and obese youth has been found to be associated with lower self-esteem, decreased physical activity, and elevated depression, anxiety, suicidality, and body dissatisfaction. Among the socio-cultural factors affecting children’s weight-related attitudes, greater media use, in particular magazine and videogame use, is significantly correlated with more negative reactions to obese girls and boys among 10–13 years old youngsters [64].

#### Psychiatric and psychological disturbances

Childhood obesity per se is not considered a behavioral or psychiatric disturbance [65,66], and most overweight young individuals do not show significant psychiatric morbidity [67]. Accordingly, in most studies of non-clinical overweight populations, the psychological condition of the youngster is found to be within normal ranges of healthy behavior [16,54,68,69], and overweight is usually not associated with elevated depression and anxiety [38,51].

Still, in several community-based studies [61,70], overweight boys and girls have been more likely to report the presence of emotional problems as compared with normal-weight adolescents, already at a very young age (3–5 years) [71] This recent study, conducted in the UK, has shown that by the ages of 3–5 years, obese boys have more conduct problems, hyperactivity, inattention problems and peer relation problems than normal weight children. Interestingly, obese girls of these respective ages present only with more peer relation problems in comparison to normal weight girls, suggesting obese boys to be at a greater risk for the development of emotional and behavioral problems already at an early age [71]. Lastly, another study has found that 23% of non-referred obese youngsters (mean age around 14 years) meet criteria for at least one mental disorder (in comparison to 37.5% of youngsters referred for overweight treatment; [72]).

In contrast to community populations, elevated rates of psychopathological disturbances, primarily depression, anxiety, somatoform disorders and eating disorders, are usually reported in clinical populations of overweight youngsters [16,24]. It is not surprising thus to find that in many studies [73], although not all [20], clinical samples of obese children have a less favorable quality of life and self-esteem than community samples. One explanation for the difference between referred and non-referred obese youngsters is that the former are significantly more overweight [72]. Putatively, neuroendocrine changes associated with stress, depression, and anxiety in clinically-referred obese children may cause metabolic changes that further increase the severity of obesity in these populations [16]. Research has shown that between 30-60% of overweight children aged 5 to 18 years referred for treatment show at least one internalizing or externalizing mental disorder [16,24,72]. This rate is higher in comparison with children with many other chronic physical disorders [24].

Specifically, between 30-50% of clinical samples of obese children and adolescents show moderate to severe depression, and around a third have high levels of anxiety [74]. Conversely, latency children and adolescents suffering from depression and anxiety are at greater risk to develop elevated BMI [75], including obesity [76], in later life in comparison to healthy controls, although other studies have not found such an association [77]. Depression in obese children is associated with the magnitude of BMI in preadolescent girls but not in boys, this relationship likely explained by an excess of overweight concerns among girls [45]. In addition, overweight boys and girls have been found to engage in significantly more unhealthy risk-taking behaviors, and to experience psychosocial distress to a greater extent than their non-overweight peers [63]. In particular, overweight girls may attempt suicide to a greater extent than normal-weight girls.

Psychopathology in overweight children is associated with gender (girls report more problems than boys), ethnicity (African-American youngsters report more problems than Caucasians), peer teasing, obesity-induced distress, body dissatisfaction and parental overweight and overall psychopathology [24,44,63,78]. Interestingly, actual BMI is found less influential than these parameters in inducing psychological disturbances in obese and overweight youngsters. Reduction of physical activity may also mediate in the association of depression with childhood overweight [79].

#### Behavioral problems

Behavioral problems are also found to a greater extent in overweight youngsters compared with normal-weight peers, particularly in prepubertal children [52]. Moreover, youngsters with disruptive disorders are found to have a higher BMI than subjects without disruptive disorders [75], and behavioral problems during childhood and early adolescence may be associated with a greater risk to develop overweight and obesity in early adulthood [80-82]. These associations remain consistent also after adjusting for a variety of parental characteristics (i.e., demographics and life style), child dietary patterns, family meals, television watching, and participation in sports and exercise during childhood (81). For the most part, the behavioral problems of overweight children do not reach the intensity of full-blown behavioral disorders. Nevertheless, some studies have shown greater rates of full blown attention deficit hyperactivity disorder (ADHD) in obese clinically-referred school-age children vs. normal-weight children of similar ages [82-85]. Furthermore, overweight children show a greater preponderance of ADHD symptoms of both inattention, for example deficits in set shifting and attention abilities, and impulsivity in comparison to normal-weight youngsters [86,87].

Several lines of investigation have been suggested to account for the association of ADHD during childhood with overweight and obesity in adolescence or young adulthood.

1. More children with ADHD have a BMI that is greater than the mean BMI for their respective age in comparison to children with no ADHD [88].

2. A greater prevalence of ADHD has been found in obese children who also show binge eating behaviors [89]. Similarly, overrepresentation of binge eating behavior has been shown in adolescents and adults diagnosed with ADHD during childhood [90,91].

3. Binge eating behavior may be associated with impulsivity [92], considered one of the diagnostic requirements of ADHD, as well as with specific elements of impulsivity, including the urgency element [93], and the need for immediate gratification [90].

4. The presence of hyperactivity in girls with ADHD has been found to predict the development of elevated weight [94].

5. The development of overweight and obesity in children with ADHD may be associated with disturbances in several neuro-cognitive tasks that are characteristic of ADHD, partly mediated by elevated risk for binge eating. These include regulation, restraint, working memory set-shifting and perseverative modes of thinking and behavior [95,96].

6. The presence of ADHD has been found do be associated with more difficulties in the process of weight reduction and the maintenance of reduced weight in obese children [84] and adults [97].

#### Eating disorders

The lifetime rates of eating disorders (EDs), mainly bulimia nervosa (BN), binge eating disorder (BED), and/or ED not otherwise specified (ED-NOS), is significantly greater in clinical samples of overweight youngsters compared with population-based overweight groups [72,98,99]. Moreover, clinically-referred obese patients with childhood-onset obesity have a significantly higher lifetime prevalence of EDs in general, and BN in particular, than adult-onset obesity patients [100]. Still, although psychological traits associated with disordered eating, for example pursuit of thinness and body dissatisfaction, appear among community samples of obese patients, being close in this respect to young ED patients, these obese youngsters do not usually show clinically full-blown EDs [101]. Furthermore, if an overweight youngster has an ED, its course and outcome are less favorable if the ED develops in the context of premorbid normal weight, in comparison to premorbid overweight [102].

Although obesity per se is not classified as an ED, it shares important psychosocial antecedents of EDs. Accordingly, peer teasing, disturbed self- and body-esteem, and self-directed and naturalistic dieting may be all precursors for the development of both disturbances [5,77,103]. Furthermore, elevated weight may increase the risk for the development of an ED [62], conversely, disturbed eating, in children in particular binge eating, may predict an increase in body fat [77]. It is, thus, not surprising that prevention programs targeted for both problems are similar in many cardinal aspects [62], and programs aimed toward reducing the risk of one, may often improve also the condition of the other [104].

#### Binge eating disorder (BED)

BED is defined as a subcategory of the ED-NOS diagnosis in which uncontrolled binge eating episodes occur at least twice a week for a period of no less than three consecutive months, with no evidence of weight-reduction compensatory behaviors [105]. Two patterns of BED have been identified in both adults and adolescents, in that the onset of binge eating may occur either before or after the onset of dieting. Whereas in the dieting-associated BED subtype, binge eating usually appears in late adolescence, non-dieting BED may appear as early as 11–13 years of age [64,106-108].

BED is currently considered a major clinical problem in overweight individuals [51,109], as 20–40% of severely obese adults [106,110] and adolescents [51,68,98,111-113] seeking treatment report significant binge eating symptomatology. The prevalence of BED and sub-threshold BED among adult overweight individuals in the general community is much lower (5-8%) [114,115]. Although the findings for children and adolescents are still inconclusive [106], some studies have shown that up to 5.3% of overweight children 6–10 years of age may be diagnosed with BED [116].

Studies that have associated binge eating only with eating a large amount of food have shown that boys report more binges and recurrent bingeing behaviors, but girls are more embarrassed and self-critical about their binges [117]. By contrast, studies that define binge eating as including loss of control over eating as well, have found a greater extent of bingeing behaviors among girls, in keeping with findings in adults [118-122]. With respect to age, binge eating has been found to increase with age for Caucasian youngsters and decrease with age for African-American youngsters, independent of gender [123]. Compensatory weight reduction purging and non-purging restricting behaviors occur only infrequently in overweight youngsters.

For both children and adolescents, a personal and family history of obesity likely increases the risk for the development of binge eating in the context of adverse childhood experiences, problematic family relationships, maladaptive parental eating-related attitudes, weak social support, and elevated psychiatric morbidity (particularly depression, but also emotional disinhibition) [124,125]. It is not yet clear whether binge eating or BED in childhood and adolescence continuous to BED in adulthood [106].

The implications of binge-eating behavior have been repeatedly investigated in overweight adults, but only a few studies have evaluated this behavior in overweight children and adolescents. The presence of binge eating behavior in overweight individuals signifies the likelihood of greater eating-related and non-eating related lifetime morbidity in comparison to non-binge eating overweight, although an inclination towards more severe overweight has been found only in adult [126] but not in adolescent binge eaters. Non-bingeing severely overweight adults [1] and adolescents [51] report relatively few psychological difficulties, although they do score low on self-esteem, and may show considerable social difficulties. By contrast, overweight children and adolescents with binge eating behavior and BED, characterized by loss of control over eating above and beyond overeating, show problematic eating behaviors, particularly eating in the absence of hunger and in secrecy [46,125,127]. These youngsters eat significantly more, have greater weight and shape concerns, as well as significantly elevated levels of depressive and anxiety symptoms and significantly lower self-esteem, in comparison to non-bingeing overweight youngsters [46,51,116,128]. In addition, the greater the severity of bingeing behavior among overweight youngsters, the more are these youngsters at risk to develop depression, anxiety, and self-and body esteem problems [51,128].

Both adult and adolescent overweight binge eaters do not usually compensate for bingeing episodes by restricting food intake between episodes [54], and loss of control over eating in childhood binge eating is usually not associated with dieting [116]. Dieting behavior is usually not a necessary factor for the development of binge eating in overweight children [106], whereas adult overweight binge-eating individuals do diet more frequently than overweight non-binge eaters. From a different perspective, studies in adults have shown that BED and BN represent different syndromes on a continuum of disturbed eating that are likely associated with different etiological factors [106]. Accordingly, patients with BN show greater eating related and non-eating related psychopathology than BED patients [129]. This phenomenon has not been investigated yet in adolescents, but apparently BED in youngsters does not develop into BN in later life [106].

Binge eating behavior can be identified early in childhood [116,120], is relatively frequent among young overweight individuals, and can lead to considerable emotional distress and psychiatric morbidity [46,106,128]. Nevertheless, although binge eating is defined identically in children, adolescents and adults, converging evidence indicates that it is difficult to systematically diagnose BED in young children with accepted adult criteria [105,130]. Factors considered of particular relevance for this diagnostic ambiguity are the inclination of children either not to understand the meaning of loss of control when it comes to binge-eating, or to frankly deny such behaviors when asked, out of shame and embarrassment [46,109,127]. Moreover, parents also tend to deny binge-eating in their children for similar reasons. Large scale community studies applying broad, less stringent and developmentally-appropriate criteria for the characterization of BED (for example, inclusion of food seeking in the absence of hunger, and sneaking or hiding food), using age appropriate interview techniques for both children and parents, are required to improve the characterization of BED in younger age groups [106,120,130].

### Parental influences and attitudes

The likelihood of the family to be involved in the predisposition to obesity in their children and in the well-being of their obese children may be associated with a host of familial variables. These include the knowledge and involvement of the parents in the selection of food, control over food and the patterns of eating at home (e.g., the provision of healthy food, the insistence on breakfast, and the family’s meals patterns), the acting of the parents as role models and their overall impact on healthy eating and physical activity, and issues related to family dieting, satisfaction with one’s body, and weight teasing at home [131-133].

Another important factor in the well-being of obese children relates to the influence of parental psychopathology and distress on the emotional condition of the overweight child. For example, maternal anxiety may predict the overweight child’s severity of depression and anxiety (134). Still, the presence of adequate coping strategies in the child may considerably reduce the impact of the mother’s mental state on his/her well-being [134]. In addition, child neglect has been found a specifically important factor in the predisposition to obesity at young ages [135].

Thirdly, several parenting styles have been found more prevalent than others in families of obese children in comparison to normal weight children [133]. Parenting style is defined as the combination of attitudes and the emotional climate created by parents through which parental behaviors or practices are expressed. The parenting style defined as authoritarian seems to be specifically prevalent in families of obese children [133]. This style is high in parental demanding and control and low in responsiveness, i.e. low in fostering individuality and self-assertion. As such, it may interfere with teaching the child how to chose the appropriate food and regulate food choice. In addition, parenting styles defined by low demanding and high responsiveness (permissive) and by low demanding and low responsiveness (neglectful) increase the odds of having an overweight child [136]. By contrast, the authoritative parenting style, which is high in both demanding and responsiveness, sets the structure for adequate food choice at home, and supports the child in learning how to handle effectively issues related to food choice both at home and outside of home. As such, it has been found prevalent in families of normal weight children and infrequent in obese children [133,136].

#### Attitudes of parents toward their child’s overweight

Parents of overweight/obese children may be either not worried [137-143] about their child’s weight, or, alternatively, be over-concerned with and critical about it [143,144]. Not surprisingly, ignoring weight may be considerable in parents of overweight children (above 80%), than in parents of obese children (less but not 20%) [138,141,145]. Furthermore, parents are more concerned about their young children being underweight than overweight [146]. Accordingly, one study has shown that more than 50% of mothers perceiving their child to be overweight or obese are not concerned about it [139].

One explanation for the inconsistencies in parental perceptions of their child’s overweight likely relates to the tendency of parents to be unaware [138], misperceive [147,148], or disconnect between the perceived and actual weight of their child [145,149]. For example, over 70% of mothers of overweight children see them as being of similar weight to their peers, as being equally or more active than other children, and as having a diet at least as healthy as their peers [150]. Parents mostly underestimate their child’s weight, with more than 50% of parents of obese and overweight children being unable to recognize when their child is overweight [138,145,151-154]. Still, in some studies, parents have been found to overestimate their overweight child’s weight [147].

The inclination for misperception is greater for boys than for girls [147]. In addition, parents of young overweight children misperceive their child’s weight to a greater extent than parents of overweight adolescents (65% vs. 51% respectively, [145]. Interestingly, parents tend to perceive their child’s weight significantly less accurately than their own [139,155]. There is some debate whether the inaccuracy in the perception of the child’s weight is related to parental overweight. Whereas some studies have found no association between parental perceptional inaccuracy and parental overweight [145,152], others have shown a significant difference in the proportion of distorted perception of shape between mothers of normal-weight children vs. those of overweight and obese children (17 vs. 87.5%, respectively, see [153]). This inaccuracy in perception is not related to socio-economic status and education level in some studies [145,152], whereas others [139] have shown greater misperception in families with lower education levels. In addition, African-American parents have been found twice as likely to underestimate as Caucasians [145]. BMI screening and feedback, may improve parental perception to some extent [145].

In addition to parental misperception about overweight, mothers may exhibit poor overall ability to estimate the way their overweight and obese children eat [137,139]. A significant difference may, thus, be found in the proportion of distorted perception of eating habits between mothers of normal-weight children vs. those of overweight and obese children (36.3 vs. 90.8%, see [153]). Eighty-four and 96% of mothers of obese and overweight children, respectively, in that study, have actually thought that their children ate right or little [153]. Most importantly, mothers’ distorted perceptions of shape and eating habits of their overweight children [153], found already during infancy [156], may become significant independent risk factors for the prediction of frank obesity in later life.

Parental attitudes toward obesity in their children may be associated with the child’ gender and age, as well as with their own ethnic background and/or level of education. For example, parents of boys [150], or of African-American descent, or with less education [157], have been shown to associate lower risk to their child’s overweight. Parents with less education are also less inclined to take action to prevent unhealthy weight gain in their children [138]. Parents of overweight children tend not to include weight in their distinction of health, and have been found to be more concerned with their child’s health than with his/her weight [158].

Unfortunately, not only parental lack of concern [153], but also their overconcern [144], may be associated with elevated BMI and adiposity in their children. Factors increasing parental weight concern include higher child BMI, less parental underestimation of child body size, and lower child health-related quality of life [141]. In other studies, parents have been found to be concerned about overweight among children and adolescents in general, but are reluctant to address it with their own children [159]. Conversely, some researchers have reported no difference in the manner in which parents of normal and overweight children estimate their weight, with both groups likely inclining toward underestimation [137,160].

One of the most important factors affecting parental attitude toward their child’s overweight is the manner in which parents perceive and are preoccupied with their own weight. Accordingly, parents are more likely to worry about their child’s potential for future overweight if they or the other parent are, or have been, overweight [137]. In addition, parents may become over-concerned with their child’s overweight as the result of problematic consultations they have had with health care professionals [161]. Lastly, although parents appreciate the role of diet and inactivity in the causation of overweight, they tend to underestimate the difficulties involved in their children’s attempts to change maladaptive eating behaviors [140,162,163].

Parents may endorse and transmit weight based stereotypes to their children [5]. Moreover, children’s’ perceptions about their own overweight have been found to be influenced to a greater extent by the manner in which their parents relate to their overweight than by their actual BMI. For example, for overweight girls, their mothers’ weight-related over-reacting, likely leading to restriction of food, and their fathers’ overt criticism about their weight, are among the factors that have the most detrimental influence on their self-perception [53,164], and well-being [165]. By contrast, lack of parental criticism with respect to the child’s overweight may be a protective factor for the child’s self-esteem [44]. The latter youngsters tend to use adaptive compensatory methods for the regulation of self-esteem, such as reducing the importance of areas in which they are less competent and increasing the importance of domains in which they perform well [37,50].

### Limitations

The main limitation of the present review is inherent in its design, as we have specifically decided to relate to open studies in addition to controlled and randomized controlled trials (RCTs). This has been done with the purpose of broadening our scope of information and providing the clinician with relevant findings that are not addressed in RCTs. Still, this can cause doubt about the validity of some of the findings described in this review.

## Conclusions

Several important findings are highlighted in the present review paper. The school and social performance of obese children and their overall quality of life are often less favorable compared to their normal weight peers. This is a multi-faceted problem, related to greater school absenteeism and overall psychosocial stress, less nutritious diet and physical activity, more behavioral problems, and less favorable neuropsychological functioning.

Not all obese youngsters show low self esteem, and the association between the extent of overweight and global self-esteem is not straightforward. By contrast, negative body esteem is usually correlated with the severity of overweight. Normal weight children tend to show negative attitudes towards their overweight peers. Obese adolescents are usually considered less popular than normal-weight teens, and overweight during childhood and adolescence may be associated with considerable societal victimization and peer teasing. Moreover, obese children themselves perceive negatively many of their own characteristics and attach them to their overweight.

In many studies, although not in all, overweight children in community-based samples do not show a greater extent of psychiatric morbidity in comparison to normal weight populations. By contrast, clinically-referred youngsters show elevated rates of depression, anxiety, behavioral disturbances, attention deficit hyperactivity disorder, and eating disorders. BED in youngsters does not usually follow a course of dieting. Its presence in overweight children increases the likelihood of overall psychopathology. BED may be difficult to diagnose during childhood, and adult criteria are often not suitable for children.

Parents may not show concern with their child’s overweight, as they tend to underestimate its severity and to misperceive what their children actually eat. In the same token, parents tend also to underestimate the difficulties involved in their children’s attempts to change their maladaptive eating behaviors. Still, other parents may be overconcerned with their child’ overweight Whereas lack of parental criticism about the child’s overweight may be a protective factor for the child’s self-esteem and overall coping and well-being, parental criticism may negatively influence the well-being of overweight children to a greater extent than their actual BMI.

### Critical comments, clinical implications and recommendations for future research

The present review sought to evaluate both the psychosocial factors potentially associated with the predisposition to childhood obesity, and those that may account for the well-being of the obese child. Not surprisingly, both sets of factors can be divided to three dimensions: the child, the family, and the overall surroundings of the child and family.

Two points are often emphasized with respect to the obese child. The first is that obese children show greater distress and more psychopathology than overweight children. The second is that obese children in community samples seem to be less affected psychologically than clinically-referred obese children. Still, there are studies showing that greater weight by itself is not always directly indicative of greater distress and psychopathology, and that other factors have to be involved. These relate, first and foremost, to greater weight concern, both of the obese child and of the family. Another factor that seems to be of considerable importance in interfering with the functioning and well being of at least some obese children, irrespective of their actual weight, is their less favorable neurocognitive constellation.

With respect to the differentiation of community and clinical samples of obese children, it is important to note that there are studies showing considerable psychological distress and elevated psychopathology of different types in community samples of obese children. Still, it is still not clear why in some community studies obese children and adolescents show greater psychopathology than normal weight youngsters, whereas in other studies there are no differences in the psychopathology of non-clinically referred obese children and their normal weight peers.

When considering the place of the family in the life of the obese child, it seems plausible that the attitudes of the family towards the child’s weight and eating and toward weight and eating-related issues in general, have a considerable role both in the predisposition to obesity and in the well-being of the obese child. Not surprisingly, a very influential societal influence on the well-being of the obese child relates to similar aspects of weight discrimination, weight teasing, and bullying.

In line with the main findings presented in the present review, we recommend that the psychological assessment of overweight/obese young individuals should be performed with age and developmentally appropriate criteria and tools. Any psychological assessment should relate to the parents’ and children attitudes with respect to overweight, and assess the children’s overall well-being psychological functioning and psychiatric condition, their strengths as well as their weaknesses.

Future research requires the design of prospective and longitudinal studies with appropriate sample sizes, age-appropriate assessment techniques, inclusion of the families and social organizations in the interventions, and adequate long-term follow-up.

It is further recommended to encourage primary health care professionals to integrate the relevant psychosocial issues described in this review paper and in others in their evaluation procedures and intervention strategies. In particular, the use of adult psychosocial assessment tools, adult psychiatric diagnoses, and adult intervention strategies should not be the practice in younger populations.

## Competing interests

None of the authors have any conflicts of interest, financial or non-financial, to disclose.

## Authors’ contributions

Both authors read and approved the final manuscript.

## Authors’ information

This paper in based on a paper published as a book chapter and approved by the publisher: Stein, D., Latzer, Y., Fennig, S., Golan, M., Gur, E., Levin-Zamir, D., Zuberi, E., Edmunds, L., Speisser, P., & Hochberg, Z. (2011) Psychiatric, psychological, & familial parameters in childhood obesity. In Vinai Dott Piergiuseppe (Eds). *Pathways to obesity and main roads to recovery*. New York: Nova publisher (Pp: 59–82).
